# A Selective Cortico‐Limbic Network Organizes Behavior During Reward Seeking Under Threat

**DOI:** 10.1002/jnr.70150

**Published:** 2026-08-06

**Authors:** Rodrigo O. Sierra, Leticia Ramirez‐Lugo, Elizabeth Illescas‐Huerta, Axel R. Bolaños, Francisco Sotres‐Bayon

**Affiliations:** ^1^ Psychology Program Universidad Ean Bogotá Colombia; ^2^ Institute of Cellular Physiology—Neuroscience National Autonomous University of Mexico (UNAM) Mexico City Mexico; ^3^ Department of Neuroscience University of Florida Gainesville Florida USA

**Keywords:** basolateral amygdala, insular cortex, motivational conflict, nucleus accumbens, prelimbic cortex, risk assessment

## Abstract

To obtain rewards, animals must select actions while facing threats, often under competing appetitive and defensive drives and with uncertainty about harm. While neural circuits controlling isolated threats or rewards are well characterized, it remains unclear which specific cortical and subcortical nodes are causally necessary to organize behavior during motivational conflict. Here, we used the step‐down avoidance‐mediated conflict (SDAmC) task in adult male rats, which quantifies avoidance, risk assessment, and reward approach within the same session. We performed pharmacological inactivation across eight candidate structures implicated in valence and action selection: prelimbic (PL) and infralimbic (IL) cortices, lateral orbitofrontal cortex (lOFC), anterior (aIC) and posterior (pIC) insular cortices, lateral habenula (LHb), basolateral amygdala (BLA), and nucleus accumbens (NAc). Inactivation revealed a precise anatomical dissociation within this network. Silencing PL or pIC facilitated approach behavior during conflict, but with dissociable effects on risk assessment. In contrast, BLA inactivation induced a broader behavioral disinhibition evident even in non‐conflict conditions, whereas NAc inactivation disrupted the temporal organization of approach, yielding a fragmented behavioral phenotype. Notably, inactivation of IL, lOFC, aIC, and LHb did not alter conflict resolution in this paradigm. Together, these findings identify a selective cortico‐limbic network in which PL and pIC are necessary for limiting approach under conflict, BLA contributes to avoidance expression across motivational states, and NAc contributes to approach‐related behavioral organization under conflict, constraining the set of brain regions that are necessary to organize behavior when reward seeking competes with threat.

## Significance Statement

In threatening contexts, animals must resolve motivational conflict by integrating defensive and appetitive drives. Although threat and reward circuits have been well studied, the neural nodes that organize avoidance, risk assessment, and approach during conflict remain poorly defined. Here, we used a validated conflict paradigm and pharmacological inactivation to test multiple cortical and subcortical candidates. Motivational conflict depends on a restricted subset of structures: the prelimbic cortex and posterior insula are necessary for limiting approach under conflict, the basolateral amygdala contributes to avoidance expression across motivational states, and the nucleus accumbens contributes to goal‐directed behavior under conflict. Other regions showed no detectable effects under the present SDAmC conditions. This study identifies a constrained set of regions necessary for organizing motivated behavior under threat.

## Graphical abstract

In the SDAmC task, rats step down from safety and cross a threat‐associated context for saccharin. PL limits approach, pIC supports risk assessment, BLA supports avoidance across motivational states, and NAc supports goal‐directed behavior under conflict; IL, lOFC, aIC, and LHb showed no detectable effects. AI‐assisted artwork; author‐designed and validated.

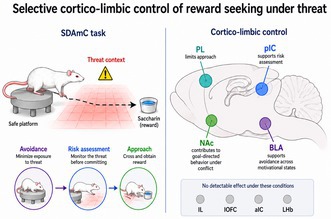

## 1 Introduction

Animals routinely face situations in which approaching a reward requires entering a potentially harmful context. Such approach‐avoidance conflicts constitute a distinct form of decision making in which appetitive and defensive motivational systems, supported by partially distinct neural circuits for threat processing (LeDoux 2000; Maren 2001; Tovote et al. 2015) and reward seeking (Berridge and Robinson 1998; Everitt and Robbins 2005; Floresco 2015), must be integrated to select a single course of action. Classical behavioral theory formalized this dilemma as competing approach and avoidance gradients (Miller 1944). Subsequent conflict paradigms operationalized it by placing reward seeking under the threat of punishment, such as punished responding and punished drinking procedures (Paterson and Hanania 2010; Vogel et al. 1971). Across species and behavioral tasks, conflict is expressed not simply as suppression of approach, but as a structured sequence of behaviors that includes hesitation, risk assessment, and action commitment. Sensitivity to anxiolytic manipulations highlights the translational relevance of these behaviors (McNaughton and Corr 2014). Importantly, approach‐avoidance conflict is not reducible to threat or reward alone but emerges when competing motivational values must be resolved under uncertainty (Calhoon and Tye 2015; Tye 2018).

Despite extensive research on the neural circuits underlying threat and reward, it remains unclear how specific cortical and subcortical nodes contribute to the internal organization of behavior during conflict. Medial prefrontal regions, including the prelimbic and infralimbic cortices, regulate defensive expression and its suppression across threat‐learning and avoidance paradigms (Bravo‐Rivera et al. 2014; Sotres‐Bayon and Quirk 2010; Vidal‐Gonzalez et al. 2006), whereas the orbitofrontal cortex supports value‐based action selection when outcomes involve competing costs and benefits (Orsini et al. 2015). The insular cortex links the interoceptive state to aversive valuation and motivational drive (Gehrlach et al. 2019), and subcortical structures, such as the basolateral amygdala and nucleus accumbens, play central roles in assigning motivational significance and guiding goal‐directed behavior (Stuber et al. 2011). In addition, the lateral habenula has been implicated in negative value signaling and aversive bias during decision making under uncertainty (Shabel et al. 2014). However, much of this literature derives from tasks that isolate threat or reward, leaving unresolved which of these regions are causally required to organize the sequence and coupling of avoidance, risk assessment, and approach that define motivated conflict. Establishing node‐specific necessity, including both positive and negative contributions, is therefore critical for constraining circuit‐level models of conflict behavior (Choi et al. 2019; Friedman et al. 2015).

To address this issue, we used the step‐down avoidance–mediated conflict (SDAmC) task, a paradigm that dissociates avoidance, risk assessment, and approach within a single behavioral episode and has been previously validated pharmacologically (Illescas‐Huerta et al. 2021). In SDAmC, rats must decide whether to descend from a safe platform onto a shock‐associated grid to obtain a palatable reward, allowing a direct comparison between conflict and non‐conflict conditions by manipulating motivational state (thirsty or not). This design isolates behavioral components that are genuinely conflict‐dependent rather than reflecting reward seeking or threat memory alone. To establish causal necessity at the regional level, we used pharmacological inactivation, examining its contribution across multiple cortical and subcortical regions implicated in threat, valuation, interoception, and motivation, including the prelimbic and infralimbic cortices, lateral orbitofrontal cortex, anterior and posterior insular cortex, lateral habenula, basolateral amygdala, and nucleus accumbens (Bravo‐Rivera et al. 2014; Floresco 2015; Gremel and Costa 2013; Moorman and Aston‐Jones 2015; Parkes et al. 2015; Ramirez‐Lugo et al. 2016; Stopper and Floresco 2014; Velazquez‐Hernandez and Sotres‐Bayon 2021; Wassum and Izquierdo 2015). By quantifying both the magnitude and the internal organization of behavioral components, we identify a selective subset of regions that exert dissociable control over risk assessment and action selection during conflict. These findings define a constrained functional architecture in which a selective subset of cortico‐limbic hubs organizes behavior when reward seeking occurs under threat.

## 2 Materials and Methods

### 2.1 Subjects

All experimental procedures were approved by the Institutional Animal Care and Use Committee of the Universidad Nacional Autónoma de México (UNAM) and were conducted in accordance with the National Ministry of Health guidelines for the care and use of laboratory animals. A total of 208 adult male Wistar rats (Instituto de Fisiología Celular, UNAM breeding colony), 2–3 months old and weighing 250–300 g at the start of the experiments, were used. Rats were housed individually in polyethylene cages to monitor water consumption, handled daily, and maintained on a 12‐h light/dark cycle. All experiments were performed during the light phase. To maintain stable motivation to seek water, rats assigned to the conflict condition were water‐restricted to 12 mL/day (6 mL in the morning and 6 mL in the afternoon). Rats assigned to the non‐conflict condition had *ad libitum* access to water, except during specific training phases, as described below, to ensure task acquisition.

### 2.2 Surgery

Rats were anesthetized with isoflurane (5% induction, 2%–3% maintenance) and placed in a stereotaxic frame (Kopf Instruments). Stainless‐steel guide cannulae (23‐gauge) were bilaterally implanted targeting the prelimbic cortex (PL; AP +3.0, ML ±0.6, DV −3.0), infralimbic cortex (IL; AP +3.0, ML ±3.1, DV −3.8; 30° angle), anterior insular cortex (aIC; AP +2.7, ML ±3.9, DV −5.2), posterior insular cortex (pIC; AP −1.8, ML ±6.2, DV −6.2), lateral orbitofrontal cortex (lOFC; AP +3.7, ML ±2.6, DV −3.6), basolateral amygdala (BLA; AP −2.8, ML ±5.0, DV −7.4), nucleus accumbens (NAc; AP +2.5, ML ±1.5, DV −5.8), and lateral habenula (LHb; AP −3.6, ML ±0.7, DV −4.0) (Paxinos and Watson 1998). Coordinates are expressed in millimeters relative to bregma. Cannulae were secured with stainless‐steel screws and dental acrylic, and obturators were inserted to maintain patency until microinfusions. Rats were allowed at least 7 days of recovery prior to behavioral procedures.

### 2.3 Step‐Down Avoidance‐Mediated Conflict (SDAmC) Task and Behavioral Protocol

**FIGURE 1:**
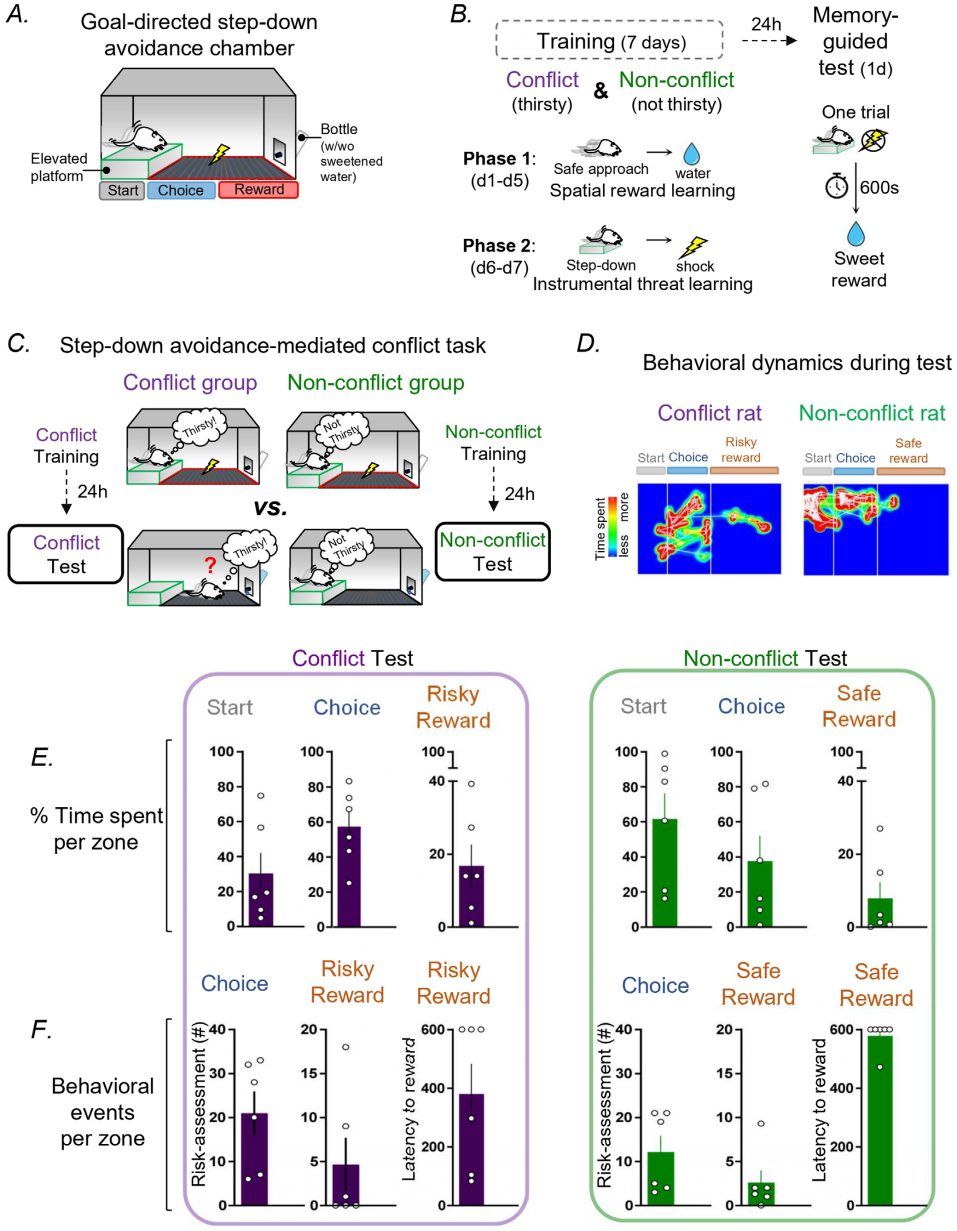
Step‐down avoidance–mediated conflict (SDAmC) task and behavioral measures. (A) Schematic of the SDAmC apparatus showing the start zone (elevated platform), choice zone (proximal grid), and reward zone (distal grid containing 0.1% saccharin). (B) Experimental design and timeline. Rats underwent reward conditioning in Phase 1 (Days 1–5), followed by threat conditioning in Phase 2 (Days 6–7; 0.5 mA shock delivered upon stepping down and terminated upon return to the platform). On the test day (Day 8), animals were tested without shock for up to 600 s under either conflict (water‐restricted; thirsty) or non‐conflict (sated) conditions. (C) Test conditions highlighting the motivational manipulation that distinguishes conflict (water‐restricted) from non‐conflict (sated) performance. (D) Representative movement heatmaps illustrating spatial occupancy during test sessions. (E, F) Quantification of time spent in each zone (E) and behavioral measures, including step‐down latency and risk assessment events (F). Conflict group, *n* = 6; non‐conflict group, *n* = 6. Data are expressed as mean ± SEM. Individual data points are represented by open circles.

Behavioral testing was conducted in a modified step‐down avoidance chamber (50 × 25 × 25 cm) housed within a sound‐attenuating cubicle (Figure 1A). The apparatus consisted of a “Safe Zone” (elevated acrylic platform; 20 × 23.5 × 6 cm) and a “Threat Zone” (stainless‐steel grid floor; 30 × 25 cm) connected to a shock generator (Coulbourn Instruments). A removable acrylic sliding door separated the platform from the grid floor. On the wall opposite to the platform, a bottle containing 0.1% saccharin served as the reward. Between sessions, the apparatus (grid, floor, and walls) was cleaned with soapy water and 70% alcohol, then dried with paper towels. For behavioral quantification, the apparatus was divided into a start zone (platform), a choice zone (proximal grid adjacent to the platform), and a reward zone (distal grid containing the saccharin bottle).

The protocol was conducted across 10 consecutive days and consisted of habituation, reward conditioning, threat conditioning, and a conflict test (Figure 1B). During habituation (two sessions), water‐restricted rats were placed on the platform for 5 min with the door closed, after which the door was opened and animals explored the apparatus for 10 min; no shock or reward was delivered. During Phase 1 (reward conditioning, Days 1–5), water‐restricted rats were placed on the platform and, after 5 min, were allowed access to saccharin; step‐down latencies were recorded daily. During Phase 2 (threat conditioning, Days 6–7), rats maintained on the water‐restriction schedule were placed on the platform; upon stepping down onto the grid with all four paws, a mild footshock (0.5 mA) was delivered continuously until the rat returned to the platform. On the test day (Day 8), no shocks were delivered, and sessions lasted up to 600 s. Conflict animals were tested under water restriction (thirsty), creating conflict between reward approach and avoidance of the shock‐associated grid. Non‐conflict animals were provided *ad libitum* water for 24 h prior to testing (sated), minimizing appetitive motivation. All animals underwent the same reward and threat conditioning procedures under water restriction. The non‐conflict condition was introduced only at test by providing ad libitum water access before the session.

### 2.4 Drug Infusion

Bilateral microinfusions were performed 15 min prior to the test session (Day 8) or the saccharin intake test. To inactivate target regions, a cocktail of the GABAA receptor agonist muscimol (250 ng/μL) and the GABAB receptor agonist baclofen (250 ng/μL; Sigma‐Aldrich), dissolved in sterile saline, was infused, while control animals received saline vehicle. Infusions were delivered through 30‐gauge injectors extending 1 mm beyond the guide cannulae and connected to 10 μL Hamilton syringes via polyethylene tubing. A total volume of 0.3–0.5 μL per hemisphere (depending on target region) was infused at 0.4 μL/min using a microinfusion pump. Injectors were left in place for an additional 1 min to allow diffusion.

### 2.5 Saccharin Intake Test

To control for nonspecific effects on fluid consumption, an independent sample of water‐restricted rats was tested in the home cage. Animals were presented with 0.1% saccharin for 30 min, and consumption was quantified by bottle weight before and after the session.

### 2.6 Histology

After behavioral testing, rats were deeply anesthetized with an overdose of pentobarbital and perfused transcardially with 0.9% saline followed by 4% paraformaldehyde. Brains were removed, post‐fixed, and cryoprotected in 30% sucrose. Coronal sections (40 μm) were cut and Nissl‐stained (cresyl violet) to verify cannula placements. Only data from animals with injector tips located within the intended target boundaries were included in statistical analyses.

### 2.7 Data Collection and Behavioral Analysis

Behavior was recorded with an overhead digital video camera and analyzed using ANY‐maze (Stoelting). Automated tracking used three body points (head, center, tail). Zone occupancy was determined using the center point for start and reward zones and the head point for the choice zone (entry threshold: 10 mm) to register risk assessment‐related probes. Step‐down latency was defined as the time from door opening to placement of all four paws on the grid (choice zone entry). Time spent in start, choice, and reward zones was quantified for each session and expressed as both duration and percentage of total session time. Risk assessment events were defined as body elongation from the platform toward the grid followed by retraction and were quantified using head‐point entries ≥ 10 mm into the choice or reward zone. Movement heatmaps were generated from head coordinates to visualize exploration patterns, with the color scale maximum set to 10 s for peak intensity. To evaluate task‐related movement during the conflict test, distance traveled and mean speed were extracted from ANY‐maze tracking data separately for the start, choice, and reward zones. These analyses were performed for the main regions in which inactivation altered conflict behavior: PL, pIC, BLA, and NAc.

### 2.8 Statistical Analysis

All analyses were performed in GraphPad Prism 10.0. Data were assessed for normality using the Kolmogorov–Smirnov test. Because behavioral variables did not consistently meet normality assumptions, comparisons between independent groups were conducted using the nonparametric Mann–Whitney *U* test. To characterize behavioral organization, Spearman rank correlations (*r*s) were computed for brain regions showing significant effects in the conflict condition. Correlation matrices included six variables: time spent in the start, choice, and reward zones; risk‐assessment event frequency in the choice and reward zones; and step‐down latency. To compare aggregate behavioral profiles across structures, each behavioral correlation matrix was flattened into a vector, and pairwise Spearman rank correlations were computed between vectors to summarize statistical similarity among regional behavioral profiles. These analyses describe statistical similarity, not anatomical connectivity. Data are presented as mean ± SEM, and statistical significance was set at *p* < 0.05. Zone‐resolved movement metrics were compared between vehicle and inactivation groups using Mann–Whitney *U* tests for each region and zone. These analyses were treated as task‐derived movement controls and were not used as primary behavioral endpoints.

## 3 Results

Rats were trained and tested on the SDAmC task, which dissociates avoidance, risk assessment, and reward approach using discrete spatial zones (start, choice, reward) within a single session (Figure 1A–C). Motivational state (thirsty vs. sated) produced behavioral differences in the expected direction across spatial and event‐based measures. In the initial characterization cohort (*n* = 6 per group), conflict animals (thirsty) tended to spend less time in the start zone and more time in the choice and reward zones, showed numerically more risk assessment events, and displayed shorter step‐down latencies than non‐conflict animals (sated); however, these direct between‐group comparisons did not reach statistical significance (% time in start, *U* = 7.0, *p* = 0.093; % time in choice, *U* = 25.0, *p* = 0.310; % time in reward, *U* = 26.0, *p* = 0.240; risk assessment at choice, *U* = 28.0, *p* = 0.128; risk assessment at reward, U = 14.0, *p* = 0.567; step‐down latency, *U* = 10.5, *p* = 0.182). We therefore use this cohort as an initial behavioral characterization of the SDAmC readouts, while the primary experimental tests focus on vehicle versus inactivation comparisons within each motivational condition. Together, these measures provided a structured framework to quantify how regional inactivation modifies conflict behavior (Figure 1E,F).

### 3.1 Prelimbic Cortex Inactivation Biases Conflict Resolution Toward Approach

**FIGURE 2:**
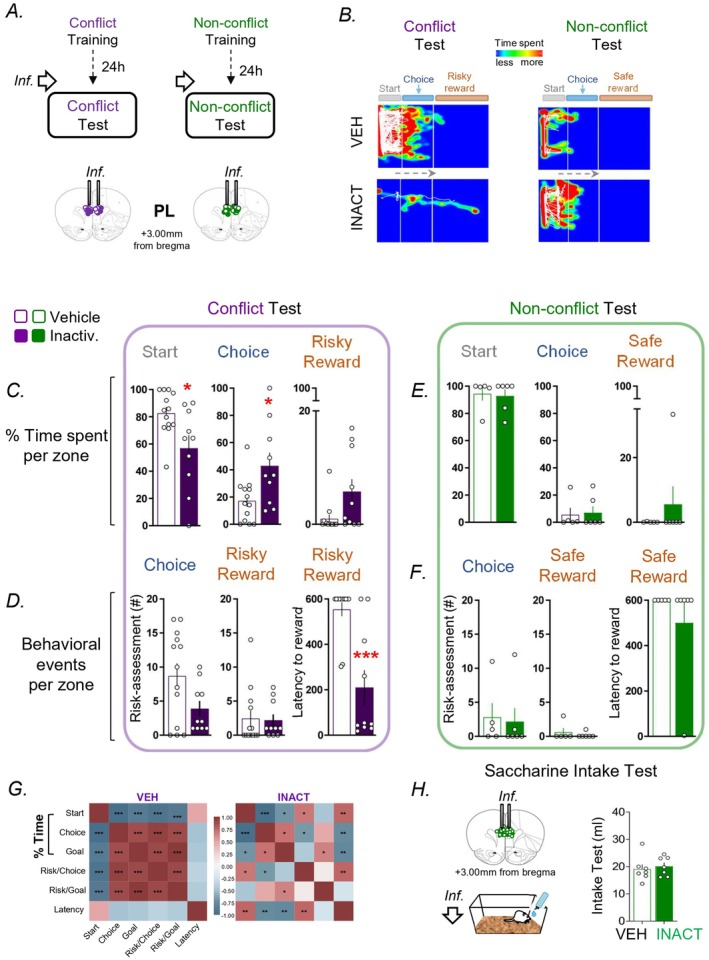
Prelimbic cortex (PL) inactivation selectively facilitates approach during conflict. (A) Cannula placements for PL and experimental timeline indicating microinfusions prior to Conflict or Non‐conflict testing. (B) Representative heatmaps during Conflict and Non‐conflict sessions under vehicle (VEH) or inactivation (INACT). (C, D) Conflict test quantification showing that PL inactivation reduced time in the start and increased time in the choice zone, with a marked reduction in step‐down latency, while risk assessment frequency was unchanged. (E, F) Non‐conflict test measures showing no detectable effects of PL inactivation under satiety. (G) Correlation matrices illustrating behavioral organization during Conflict under VEH versus INACT conditions. (H) Home‐cage saccharin intake showing no effect of PL inactivation on consummatory behavior (vehicle, *n* = 7; inactivation, *n* = 7). Conflict group (vehicle, *n* = 13; inactivation, *n* = 10); non‐conflict group (vehicle, *n* = 5; inactivation, *n* = 5). *p < 0.05; **p < 0.01; ***p < 0.001. Data are shown as mean ± SEM. Individual data points are represented by open circles.

We targeted the prelimbic cortex (PL; cannula placements shown in Figure 2A) given its established involvement in defensive control, persistent threat monitoring, and action selection under uncertainty (Bravo‐Rivera et al. 2014; Burgos‐Robles et al. 2009; Capuzzo and Floresco 2020; Sotres‐Bayon et al. 2012). Representative heatmaps are shown to visualize spatial occupancy during conflict and non‐conflict testing (Figure 2B); quantitative analyses are shown in Figure 2C–F. During the conflict test, vehicle‐treated rats displayed a strongly avoidance‐biased strategy, remaining predominantly in the start zone with limited transitions onto the grid (Figure 2B, top). In contrast, PL inactivation produced robust and selective changes: time in the start zone decreased (Mann–Whitney, *U* = 104, *p* = 0.016; Figure 2C), time in the choice zone increased (Mann–Whitney, *U* = 26, p = 0.016; Figure 2C), and step‐down latency was markedly reduced (Mann–Whitney, *U* = 17, *p* < 0.001; Figure 2D), consistent with faster commitment to enter the grid. Notably, risk assessment frequency in both the choice and reward zones was unchanged (Figure 2D), suggesting that PL inactivation facilitated approach without a parallel increase in overt risk sampling.

To test whether these effects reflected impaired threat memory retrieval or nonspecific locomotor changes, we examined a separate cohort tested under non‐conflict conditions (sated). In the absence of conflict, PL inactivation did not alter any behavioral measure (all *p* > 0.05; Figure 2E,F), indicating that PL recruitment is selectively engaged when motivational competition is present. Next, we asked whether PL inactivation altered not only behavioral outputs but also their internal coordination. Under vehicle conditions during conflict, behavioral variables showed a coherent correlational structure. PL inactivation disrupted this correlational structure: the coupling between risk assessment events in choice and reward was abolished (*r*s = 0.08, *p* = 0.828), the relationship between time in start and risk assessment in choice reversed direction (*r*s = 0.70, *p* = 0.025), and time in start became positively associated with step‐down latency (*r*s = 0.77, *p* = 0.010), an association not observed under vehicle conditions (Figure 2G). Finally, saccharin intake in the home cage was unaffected, arguing against nonspecific changes in appetitive motivation (*t*(12) = −0.478, *p* = 0.642; Figure 2H). Overall, these findings suggest that PL activity contributes to maintaining an avoidance‐biased strategy during motivational conflict and to preserving the coordination linking avoidance, risk sampling, and action initiation.

### 3.2 Inactivation of IL and lOFC Does Not Alter Conflict Behavior

**FIGURE 3:**
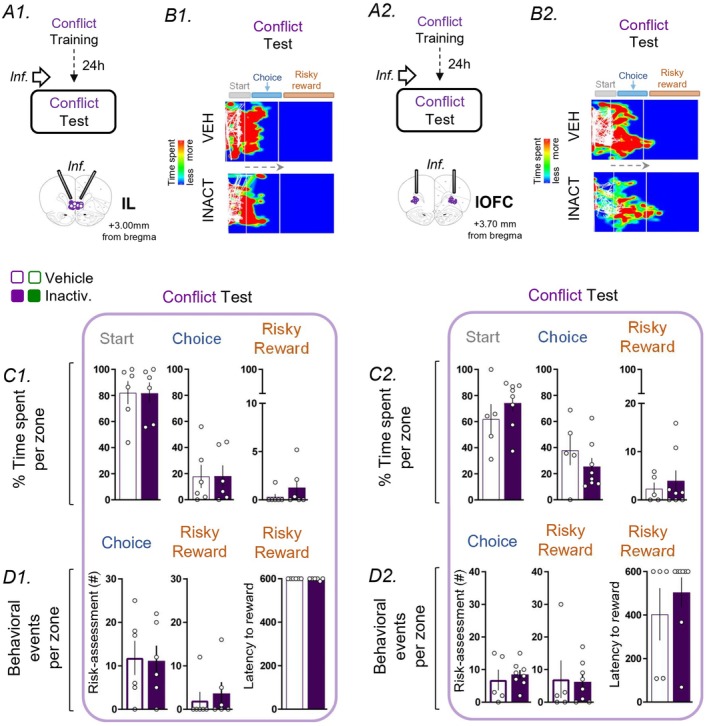
Inactivation of infralimbic cortex (IL) or lateral orbitofrontal cortex (lOFC) does not alter conflict behavior. (A1, A2) Cannula placements and timelines for IL (A1) and lOFC (A2) inactivation prior to Conflict testing. (B1, B2) Representative heatmaps showing comparable spatial occupancy patterns under vehicle (VEH) and inactivation (INACT) conditions. (C1, C2) Time spent in the start, choice, and reward zones during Conflict for IL (C1) and lOFC (C2), showing no significant differences. (D1, D2) Risk assessment measures and step‐down latency remained unchanged following IL or lOFC inactivation. IL: (vehicle, *n* = 6; inactivation, *n* = 6); lOFC: (vehicle, *n* = 5; inactivation, *n* = 8). Data are shown as mean ± SEM. Individual data points are represented by open circles.

To determine whether PL effects reflected a broader frontal cortical contribution, we tested the infralimbic cortex (IL; Figure 3A1), implicated in suppressing defensive responses (Sierra‐Mercado et al. 2011), and the lateral orbitofrontal cortex (lOFC; Figure 3A2), implicated in value‐based updating and action selection (Gremel and Costa 2013; Ramirez‐Lugo et al. 2016). Representative heatmaps show similar occupancy patterns in vehicle and inactivation conditions for both IL and lOFC during conflict testing (Figure 3B1,B2). Consistent with this, inactivation of either region did not alter conflict performance: time allocation across the start, choice, and reward zones, risk assessment frequency, and step‐down latency were comparable between vehicle and inactivation conditions (all *p* > 0.05; Figure 3C1–D2). Because IL and lOFC inactivation produced no detectable effects during conflict, these regions were not examined further in non‐conflict, saccharin intake, or correlation analyses. These data indicate that under the present SDAmC conditions, IL and lOFC inactivation produced no detectable changes in the behavioral measures quantified here, in contrast to the effects observed after PL inactivation.

### 3.3 Posterior Insular Cortex Inactivation Facilitates Conflict Resolution

**FIGURE 4:**
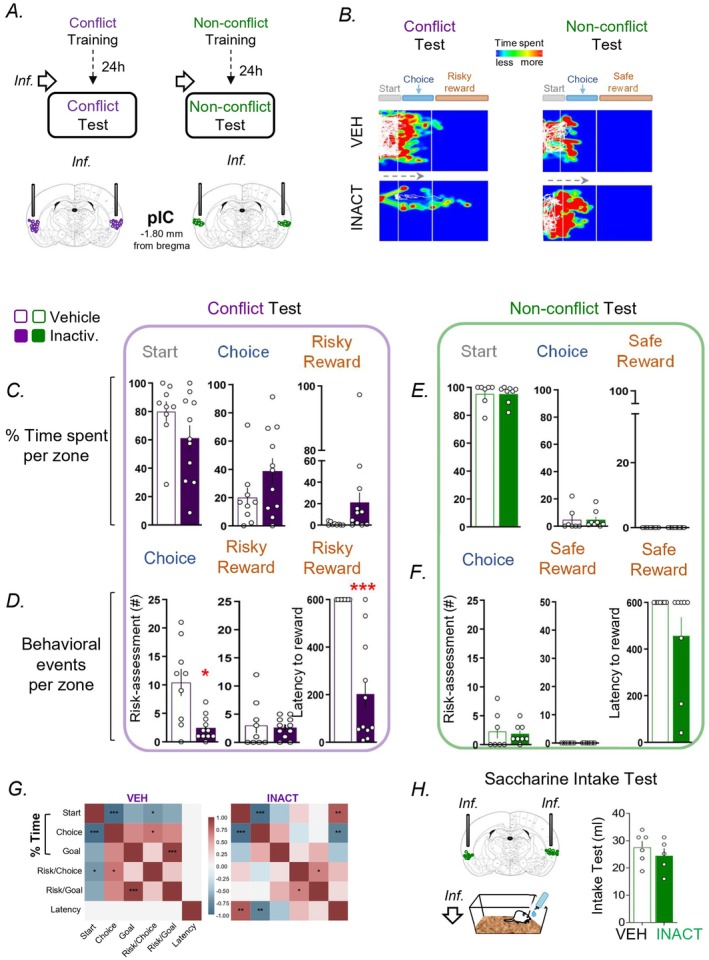
Posterior insular cortex (pIC) inactivation facilitates conflict resolution and reduces risk assessment. (A) Cannula placements for pIC and timeline indicating microinfusions prior to Conflict or Non‐conflict testing. (B) Representative heatmaps illustrating behavior during Conflict and Non‐conflict sessions under vehicle (VEH) or inactivation (INACT). (C, D) Conflict test quantification showing that pIC inactivation reduced step‐down latency and decreased risk assessment in the choice zone, with no significant change in time in the start zone. (E, F) Non‐conflict test measures showing no detectable effects of pIC inactivation under satiety. (G) Correlation matrices illustrating behavioral organization during Conflict under VEH versus INACT conditions. (H) Home‐cage saccharin intake showing no effect of pIC inactivation on consummatory behavior (vehicle, *n* = 6; inactivation, *n* = 5). Conflict group (vehicle, *n* = 9; inactivation, *n* = 11); non‐conflict group (vehicle, *n* = 7; inactivation, *n* = 8). *p < 0.05; **p < 0.01; ***p < 0.001. Data are expressed as mean ± SEM. Individual data points are represented by open circles.

The posterior insular cortex (pIC; cannula placements shown in Figure 4A) was targeted based on its role in processing interoceptive state and aversive consequences (Gehrlach et al. 2019; Livneh et al. 2017). Representative heatmaps are shown to visualize spatial occupancy during conflict and non‐conflict testing (Figure 4B); quantitative analyses are shown in Figure 4C–F. During conflict, vehicle‐treated rats showed the expected avoidance‐biased pattern (Figure 4B, top). pIC inactivation reduced step‐down latency and decreased risk assessment events at the choice zone: step‐down latency was markedly reduced (Mann–Whitney, *U* = 4.5, *p* < 0.001; Figure 4D), and risk assessment events in the choice zone decreased (Mann–Whitney, *U* = 16.5, *p* = 0.010; Figure 4D), whereas time in start zone did not differ significantly (*p* > 0.05; Figure 4C). These effects were selective to motivational conflict: in a separate cohort tested under non‐conflict conditions, pIC inactivation did not alter behavior (all *p* > 0.05; Figure 4E,F). Correlation analyses revealed altered statistical associations among behavioral measures. Under vehicle, time in start was negatively associated with choice risk assessment (*r*s = −0.70, *p* = 0.035), and reward time was strongly coupled to risk assessment (*r*s = 0.96, *p* < 0.001). After pIC inactivation, the start–risk assessment association was lost (*r*s = 0.26, *p* = 0.445), reward time–risk assessment coupling weakened (*r*s = 0.45, *p* = 0.168), and a strong positive association emerged between time in start and step‐down latency (*r*s = 0.84, *p* = 0.001), absent under vehicle conditions (Figure 4G). Saccharin intake was unchanged (*p* > 0.05; Figure 4H). Together, these results identify pIC as a critical node that constrains approach during conflict, with effects dissociable from PL by virtue of reduced risk assessment.

### 3.4 Inactivation of aIC and LHb Does Not Alter Conflict Behavior

**FIGURE 5:**
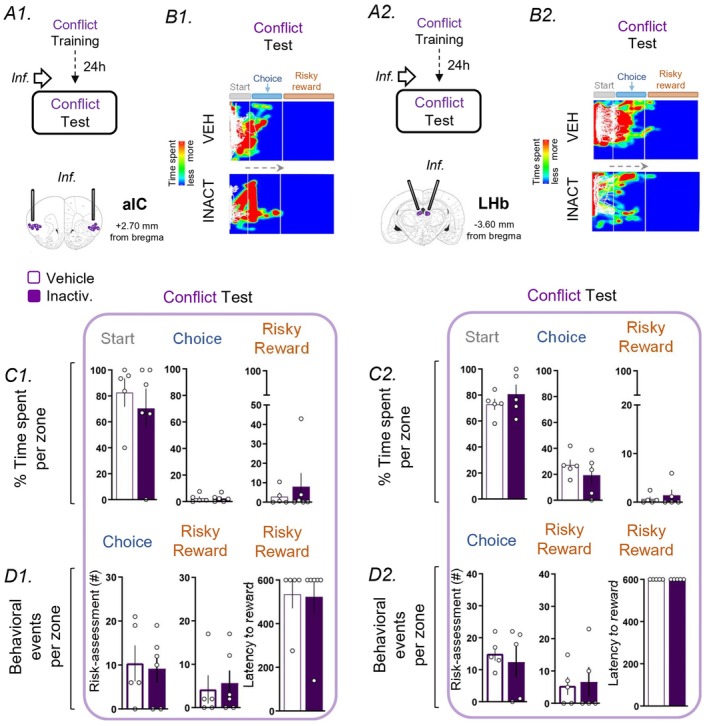
Inactivation of anterior insular cortex (aIC) or lateral habenula (LHb) does not alter conflict behavior. (A1, A2) Cannula placements and timelines for aIC (A1) and LHb (A2) inactivation prior to Conflict testing. (B1, B2) Representative heatmaps showing similar spatial occupancy under vehicle (VEH) and inactivation (INACT) conditions. (C1, C2) Time spent in the start, choice, and reward zones during Conflict for aIC (C1) and LHb (C2), showing no significant differences. (D1, D2) Risk assessment measures and step‐down latency remained unchanged following aIC or LHb inactivation. aIC: (vehicle, *n* = 5; inactivation, *n* = 5); LHb: (vehicle, *n* = 5; inactivation, *n* = 5). Data are shown as mean ± SEM. Individual data points are represented by open circles.

To further define anatomical specificity, we tested the anterior insular cortex (aIC; Figure 5A1), implicated in salience‐related control of approach (Rogers‐Carter et al. 2019), and the lateral habenula (LHb; Figure 5A2), implicated in aversive signaling and avoidance bias (Shabel et al. 2014; Stamatakis and Stuber 2012; Velazquez‐Hernandez and Sotres‐Bayon 2021). Representative heatmaps show no apparent shift in spatial distribution following aIC or LHb inactivation during conflict (Figure 5B1,B2). Consistent with this, inactivation of either region did not alter conflict behavior: spatial allocation across the start, choice, and reward zones, risk assessment frequency, and step‐down latency were comparable between vehicle and inactivation conditions (all *p* > 0.05; Figure 5C1–D2). Given the absence of primary effects during conflict, these regions were not evaluated further. These null results constrain the network identified here, highlighting an anterior–posterior dissociation within insula cortex (pIC, but not aIC) and indicating that LHb inactivation did not produce detectable changes in the behavioral measures quantified under the present SDAmC conditions.

### 3.5 Basolateral Amygdala Inactivation Induces Generalized Loss of Avoidance

**FIGURE 6:**
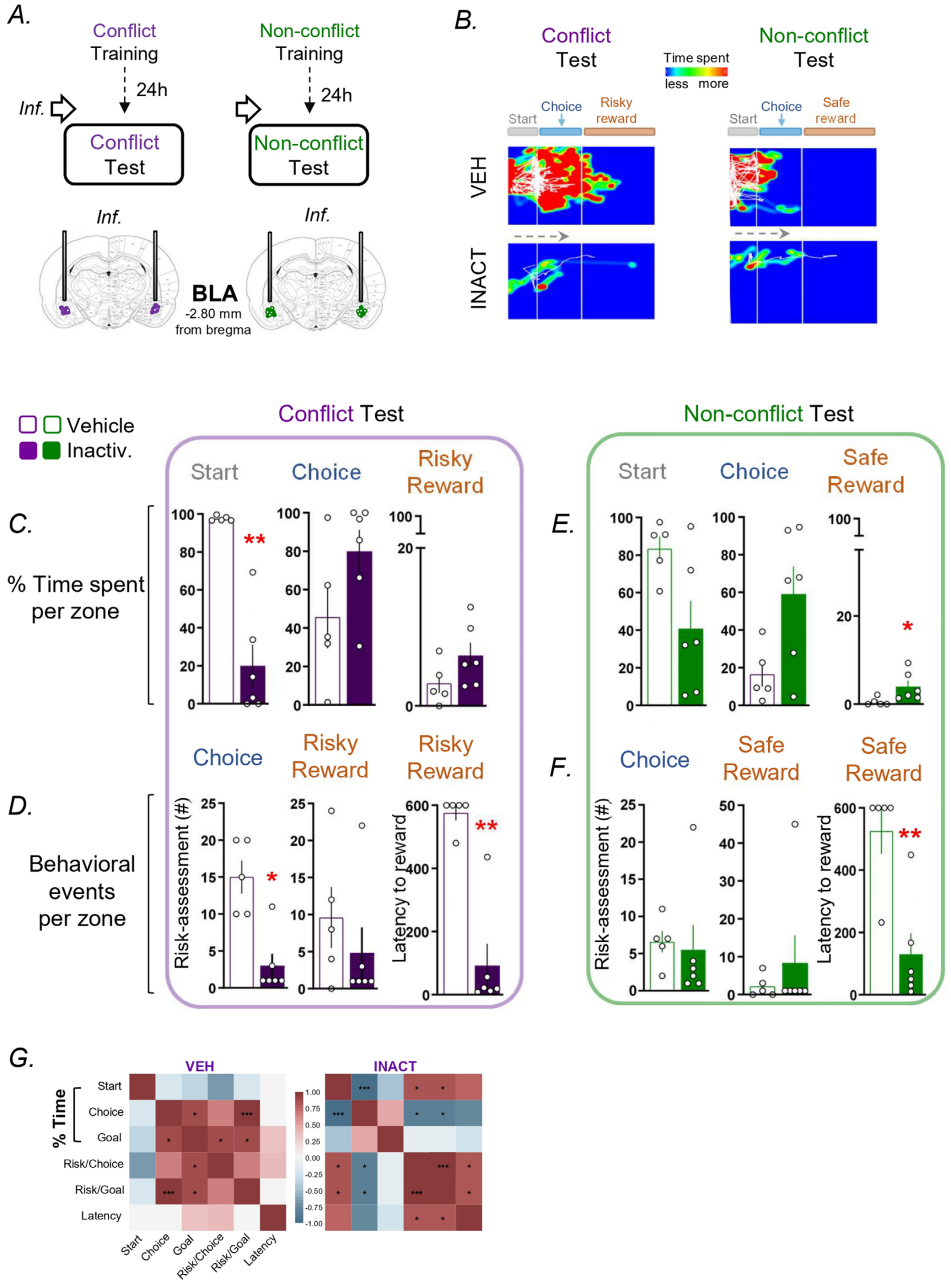
Basolateral amygdala (BLA) inactivation induces generalized loss of avoidance across motivational states. (A) Cannula placements for BLA and timeline indicating microinfusions prior to Conflict or Non‐conflict testing. (B) Representative heatmaps showing increased progression onto the grid following inactivation (INACT) during both conflict and non‐conflict sessions. (C, D) Conflict test measures showing reduced time in start, reduced risk assessment in choice, and reduced step‐down latency after BLA inactivation. (E, F) Non‐conflict test measures showing that BLA inactivation facilitated approach even under satiety, including increased time in reward and reduced step‐down latency. (G) Correlation matrices illustrating behavioral organization during Conflict under vehicle (VEH) versus inactivation (INACT) conditions. Conflict group (vehicle, *n* = 5; inactivation, *n* = 6); non‐conflict group (vehicle, *n* = 5; inactivation, *n* = 6). *p < 0.05; **p < 0.01; ***p < 0.001. Data are shown as mean ± SEM. Individual data points are represented by open circles.

The basolateral amygdala (BLA; cannula placements shown in Figure 6A) was targeted given its central role in valence encoding and threat memory expression (Beyeler et al. 2016; Blair et al. 2005; Janak and Tye 2015; Namburi et al. 2015). Representative heatmaps are shown to visualize spatial occupancy during conflict and non‐conflict testing; quantitative analyses showed that BLA inactivation reduced time in the start zone during conflict and increased time in the reward zone under non‐conflict conditions (Figure 6B). During conflict, BLA inactivation reduced time in the start zone (Mann–Whitney, *U* = 13.00, *p* = 0.043; Figure 6C), decreased risk assessment in choice (Mann–Whitney, *U* = 14.50, *p* = 0.047; Figure 6D), and reduced step‐down latency (Mann–Whitney, *U* = 9.00, *p* = 0.008; Figure 6D), consistent with facilitated approach. Unlike PL and pIC, BLA inactivation also altered behavior in the non‐conflict test: in satiated rats, time in the reward zone increased (Mann–Whitney, *U* = 3.00, *p* = 0.030; Figure 6E) and step‐down latency decreased (Mann–Whitney, *U* = 2.00, *p* = 0.015; Figure 6F), suggesting generalized loss of avoidance independent of motivational state. Correlation analyses revealed altered statistical associations among behavioral measures. Under vehicle, time in the choice zone was perfectly correlated with risk assessment in reward (*r*s = 1.00, *p* < 0.001), whereas after BLA inactivation, this relationship reversed (*r*s = −0.86, *p* = 0.029). Risk assessment events in choice and reward became perfectly coupled (*r*s = 1.00, *p* < 0.001) and were strongly associated with step‐down latency (*r*s = 0.85, *p* = 0.034), indicating altered coordination of risk sampling dynamics (Figure 6G). Overall, these findings suggest that BLA activity contributes to avoidance expression in this task, and its inactivation produces generalized facilitation of approach across motivational states.

### 3.6 Nucleus Accumbens Inactivation Alters Approach‐Related Behavior During Conflict

**FIGURE 7:**
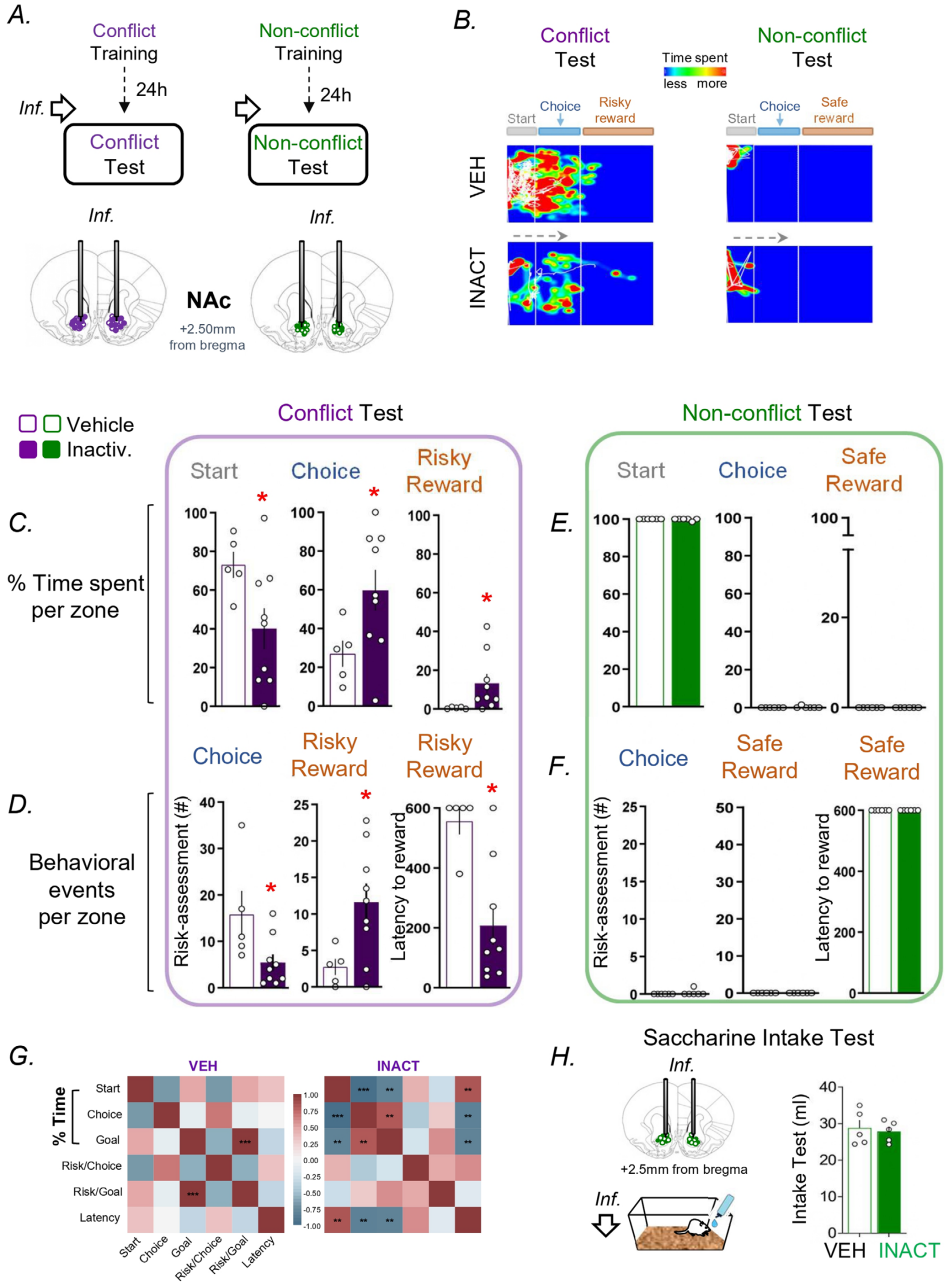
Nucleus accumbens (NAc) inactivation increases progression toward the goal while disrupting risk assessment organization during conflict. (A) Cannula placements for NAc and timeline indicating microinfusions prior to Conflict or Non‐conflict testing. (B) Representative heatmaps showing increased occupancy on the grid following inactivation (INACT) during Conflict, with minimal change under non‐conflict conditions. (C, D) Conflict test measures showing reduced time in the start zone, increased time in the choice and reward zones, reduced step‐down latency, and a mixed redistribution of risk assessment (decreased in the choice zone, increased in reward). (E, F) Non‐conflict test measures showing no detectable effects of NAc inactivation under satiety. (G) Correlation matrices illustrating behavioral organization during Conflict under vehicle (VEH) versus inactivation (INACT) conditions. (H) Home‐cage saccharin intake showing no effect of NAc inactivation on consummatory behavior (vehicle, *n* = 5; inactivation, *n* = 5). Conflict group (vehicle, *n* = 5; inactivation, *n* = 9); non‐conflict group (vehicle, *n* = 6; inactivation, *n* = 6). *p < 0.05; **p < 0.01; ***p < 0.001. Data are shown as mean ± SEM. Individual data points are represented by open circles.

The nucleus accumbens (NAc; cannula placements shown in Figure 7A) was targeted given its role in translating motivational state into goal‐directed action (Ambroggi et al. 2008; Floresco 2015; Salamone and Correa 2012; Salamone et al. 2016). Representative heatmaps show increased progression toward the grid and reward zones after NAc inactivation during conflict, with minimal change in non‐conflict conditions (Figure 7B). During conflict, NAc inactivation reduced time in the start zone (Mann–Whitney, *U* = 7, *p* = 0.042; Figure 7C) and increased time in the choice and reward zones (Mann–Whitney, *U* = 7, *p* = 0.042; and *U* = 4.5, *p* = 0.012; Figure 7C), while reducing step‐down latency (Mann–Whitney, *U* = 4, *p* = 0.011; Figure 7D). However, this progression toward the goal was accompanied by a disruption in risk assessment organization: risk assessment decreased in choice (Mann–Whitney, *U* = 6, *p* = 0.026; Figure 7D) but increased in reward (Mann–Whitney, *U* = 7.5, *p* = 0.043; Figure 7D), consistent with impaired coordination of risk sampling across spatial zones. In the non‐conflict test, NAc inactivation did not alter spatial distribution or step‐down latency (all *p* > 0.05; Figure 7E,F), and saccharin intake was unaffected (Figure 7H), suggesting that the effects are not explained by reduced motivation. The absence of a consistent increase in mean speed during the conflict task, together with the condition‐dependent nature of the behavioral effects, argues against generalized hyperlocomotion as the main explanation for the increased approach phenotype (see Figure S1). Correlation analyses revealed altered correlational patterns: under vehicle, reward time was perfectly correlated with reward risk assessment (*r*s = 1.00, *p* < 0.001), whereas after NAc inactivation this association weakened (*r*s = 0.60, *p* = 0.088). Time in the start zone was negatively correlated with time in the reward zone (*r*s = −0.83, *p* = 0.005) and positively correlated with step‐down latency (*r*s = 0.85, *p* = 0.004); time in the choice and reward zones became positively correlated (*r*s = 0.83, *p* = 0.005), and time in the reward zone became negatively associated with step‐down latency (*r*s = −0.83, *p* = 0.005) (Figure 7G). Together, these results indicate that NAc activity contributes to approach‐related behavioral measures and to the statistical associations among spatial allocation, risk assessment, and step‐down latency during motivational conflict.

Across structures, these results reveal a constrained and anatomically selective network supporting conflict behavior. PL and pIC inactivation biased performance toward approach specifically under motivational conflict, but with dissociable impacts on risk assessment, whereas BLA inactivation produced a generalized loss of avoidance evident even in non‐conflict conditions. In contrast, NAc inactivation promoted spatial progression toward the goal while disrupting the coordination of risk assessment across zones, consistent with impaired organization of goal‐directed action under threat. Finally, several candidate regions commonly implicated in valuation or aversive signaling (IL, lOFC, aIC, and LHb) showed no detectable effects under the present SDAmC conditions. We next integrate these positive and negative findings to identify the constrained set of hubs required to organize avoidance, risk assessment, and approach during reward seeking under threat.

To further evaluate whether the increased approach observed after regional inactivation could be explained by nonspecific motor changes, we analyzed task‐related movement metrics in the main regions where inactivation altered conflict behavior: PL, pIC, BLA, and NAc. Distance traveled and mean speed were quantified separately for the start, choice, and reward zones during the conflict test (Figure S1). Distance traveled was interpreted cautiously because movement across zones in SDAmC is constrained by the animal's approach–avoidance strategy. Task‐related movement was concentrated primarily in the choice zone. Regional inactivation produced zone‐specific redistribution of distance traveled, including reduced choice‐zone distance after pIC, BLA, and NAc inactivation and increased reward‐zone distance after PL, pIC, and NAc inactivation. In contrast, mean speed did not show a generalized increase across regions or zones. These zone‐resolved movement analyses indicate that inactivation altered where movement occurred within the task rather than producing a generalized increase in locomotor output. Together with the condition‐dependent behavioral effects, these findings argue against generalized hyperlocomotion as the primary explanation for the increased approach phenotype.

## 4 Discussion

**FIGURE 8:**
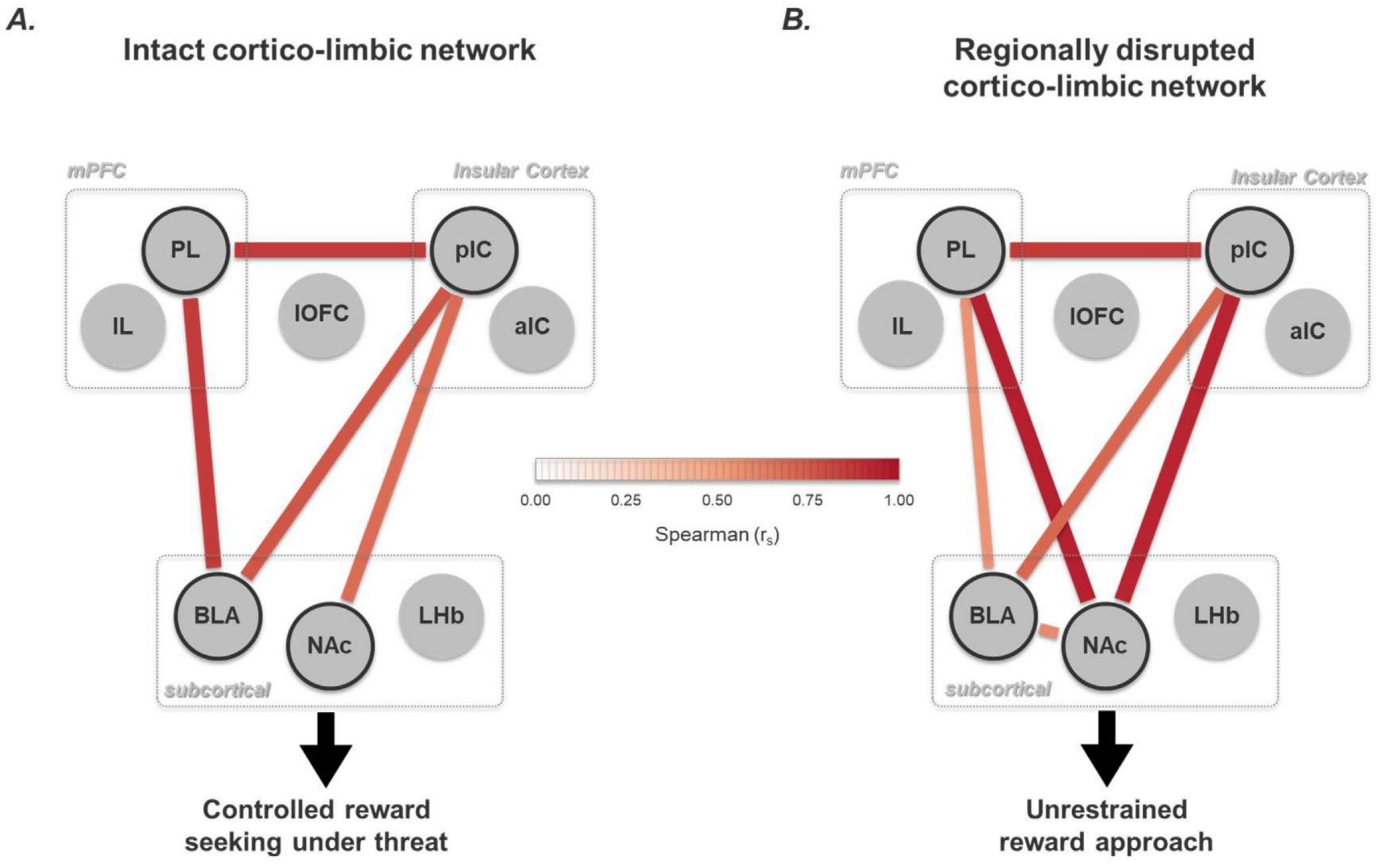
Functional network schematic comparing the vehicle and regional inactivation conditions. Schematic representation of the cross‐regional functional coupling among the main nodes implicated by the SDAmC, shown separately for the vehicle condition (A) and the regional inactivation condition (B). Circles denote regional nodes (PL, IL, lOFC, pIC, aIC, BLA, NAc, LHb), dashed boxes group nodes into cortical networks. Black‐outlined nodes highlight regions identified as critical hubs in the behavioral analyses (i.e., regions whose inactivation produced robust changes in the conflict phenotype), whereas non‐outlined/gray nodes indicate regions without a detectable behavioral impact under the present paradigm. Edges depict Spearman rank correlations (*r*s) between regional behavioral profiles (color‐coded bar) and represent statistical associations rather than anatomical connectivity. This schematic summarizes the task‐specific network configuration supporting conflict‐related avoidance, risk assessment, and the organization of goal‐directed action, and illustrates how regional inactivation reshapes coupling patterns among the key hubs.

In this study, we used a validated approach‐avoidance paradigm (Illescas‐Huerta et al. 2021) and systematic pharmacological inactivation to identify which brain regions are necessary to organize behavior during reward‐seeking under threat. Our main finding is that conflict performance depends on a constrained set of cortico‐limbic regions under the present SDAmC conditions, rather than a broad engagement of regions commonly implicated in threat or valuation. Among the eight candidate structures tested, only PL, pIC, BLA, and NAc showed detectable effects on conflict behavior, and each region contributed in a dissociable manner. PL and pIC inactivation facilitated approach selectively under motivational conflict, BLA inactivation produced a generalized loss of avoidance that extended to non‐conflict conditions, and NAc inactivation disrupted the coordination of risk assessment and approach despite increasing progression toward the reward. In contrast, IL, lOFC, aIC, and LHb did not show detectable effects under the present experimental conditions, providing an informative set of negative results that constrains mechanistic models of approach‐avoidance conflict. Together, these findings support a selective architecture in which a limited set of hubs is necessary to organize avoidance, risk assessment, and approach when reward pursuit competes with threat. These correlational patterns reflect statistical covariation rather than anatomical connectivity. A consolidated view of the positive and null effects is provided in Table 1, while a network‐level interpretation of the critical hubs is provided in Figure 8A,B.

**TABLE 1:** Summary of behavioral effects of regional inactivation in the SDAmC.

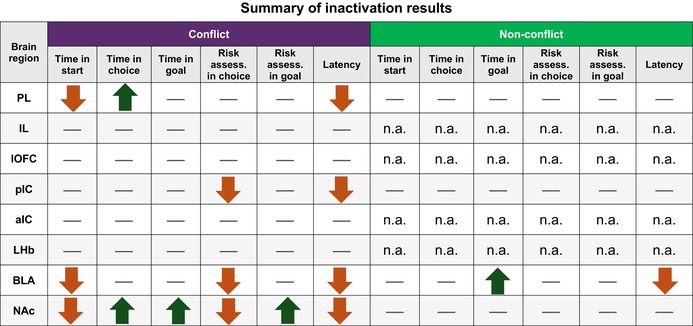

*Note:* Table summarizing the direction and specificity of behavioral changes produced by regional inactivation relative to vehicle across the SDAmC. For each region, effects are indicated separately for the conflict and non‐conflict conditions and across the three task zones (start, choice, reward), as well as the main derived measures (risk assessment at choice and reward, and latency). Upward (green) and downward (red) arrows denote significant increases or decreases relative to the vehicle, respectively. “—” indicates no detectable effect under the present paradigm, whereas “n.a.” indicates that the behavioral variable was not measurable because there was no initial conflict‐related effect during the evaluation.

### 4.1 PL Contributes to Limiting Conflict‐Specific Approach Without Increasing Overt Risk Sampling

PL inactivation biased conflict resolution toward approach, reflected by reduced time in the start zone, increased time in the choice zone, and a strong reduction in step‐down latency. These effects were selective to conflict conditions, suggesting that PL is preferentially recruited when threat and reward compete. This is consistent with convergent evidence implicating PL in defensive control, persistent monitoring of threat, and the expression of avoidance strategies (Bravo‐Rivera et al. 2014; Sierra‐Mercado et al. 2011; Sotres‐Bayon and Quirk 2010; Sotres‐Bayon et al. 2012). Importantly, PL inactivation did not increase the frequency of risk assessment, indicating that the approach bias was not accompanied by heightened overt sampling of the grid. Instead, correlation analyses showed that PL inactivation reorganized the internal structure of behavior, particularly by decoupling risk assessment across zones and altering relationships between start occupancy, risk assessment, and latency. This pattern suggests PL primarily maintains a coherent defensive strategy linking evaluation to action timing under motivational competition, rather than generating risk assessment events per se. Within the SDAmC framework, PL may therefore act as a cortical hub that stabilizes a conservative strategy, preserving hesitation and threat sensitivity when appetitive drive is high.

### 4.2 pIC Constrains Approach by Supporting Decision‐Point Risk Evaluation

pIC inactivation also facilitated approach under conflict, but with a distinct signature: a large reduction in step‐down latency accompanied by decreased risk assessment in the choice zone. These effects were absent in non‐conflict conditions and occurred without changes in home‐cage saccharin intake, arguing against nonspecific changes in motivation. The pIC has been implicated in processing aversive states and interoceptive signals that shape avoidance and threat‐related valuation (Gehrlach et al. 2019; Livneh et al. 2017). In this task, pIC appears particularly important at the decision point, where animals typically probe the grid and sample threat proximity before committing to descent. The loss of risk assessment after pIC inactivation suggests that this region supports a component of conflict behavior that is not reducible to avoidance output alone but rather reflects active evaluation under uncertainty. Notably, the dissociation between aIC and pIC is consistent with the idea that the insula is functionally heterogeneous (Gehrlach et al. 2019; Livneh et al. 2017; T. Shi et al. 2020; W. Shi et al. 2022), and that posterior subdivisions may be more directly coupled to aversive consequences and bodily state computations relevant for approach‐avoidance arbitration.

Importantly, the effects of PL and pIC inactivation are unlikely to reflect nonspecific locomotor activation or generalized increases in reward motivation. These inactivations did not alter behavior under non‐conflict conditions, did not affect saccharin consumption, and did not produce a consistent increase in mean speed during the conflict task (Figure S1), indicating that these manipulations selectively altered conflict‐dependent action selection rather than producing generalized hyperlocomotion.

### 4.3 BLA Contributes to Avoidance Expression Across Motivational States

BLA inactivation produced a qualitatively different phenotype from PL or pIC: facilitated approach during conflict was accompanied by similar facilitation in non‐conflict conditions, including increased time near the reward and reduced step‐down latency under satiety. This generalized effect is consistent with BLA's central role in threat memory expression and associative control of defensive behavior (Bravo‐Rivera et al. 2014; Burgos‐Robles et al. 2017; Hernandez‐Jaramillo et al. 2024; Namburi et al. 2015; Pickens et al. 2003; Tye et al. 2011). In SDAmC, the grid carries an instrumental threat contingency acquired during threat conditioning; weakening that association would be expected to reduce avoidance regardless of motivational state. Thus, while PL and pIC modulated how behavior is organized under competition, BLA activity appeared to contribute to the aversive constraint that defines the task. This interpretation also aligns with extensive work positioning the amygdala as a key hub for linking learned threat to behavioral output, which can then be shaped by prefrontal regulation depending on context and competing motivations (Dallerac et al. 2017; Moscarello and LeDoux 2013; Pape and Pare 2010; Sierra‐Mercado et al. 2011; Sotres‐Bayon and Quirk 2010). Free saccharin consumption was not assessed following BLA inactivation. Because BLA inactivation altered avoidance‐related behavior across both conflict and non‐conflict conditions, rather than selectively increasing reward approach during conflict, the BLA phenotype is unlikely to be explained solely by increased saccharin consumption. Nevertheless, we cannot fully exclude a contribution of altered saccharin valuation.

### 4.4 NAc Contributes to the Organization of Goal‐Directed Behavior Under Conflict

NAc inactivation increased progression toward the reward under conflict, reducing time in the start zone and decreasing step‐down latency, but produced a distinctive disorganization of risk assessment: risk assessment decreased at the choice zone yet increased at the reward zone. This mixed pattern suggests a disruption in the temporal organization of exploration and evaluation, rather than simple facilitation of approach. NAc is classically involved in translating motivational state into action selection and vigor (Ambroggi et al. 2008; Stuber et al. 2011). Within SDAmC, conflict requires not only initiating movement toward the reward but coordinating when and where risk sampling occurs as the animal advances across threat‐associated space. The reorganization of behavioral correlations after NAc inactivation supports the view that NAc contributes to coupling spatial progression with structured evaluation, enabling goal‐directed action to unfold coherently under threat. That NAc inactivation did not alter non‐conflict behavior or saccharin intake further argues against a primary deficit in locomotion or appetite, pointing instead to a role in organizing the structure of conflict‐specific action.

### 4.5 Informative Null Effects Refine the Candidate Network for Motivational Conflict

A central contribution of this study is the systematic inclusion of plausible candidate regions that did not produce detectable changes in the behavioral measures quantified under the present SDAmC conditions. IL, often associated with suppression of defensive responding and extinction‐related processes (Sierra‐Mercado et al. 2011; Vidal‐Gonzalez et al. 2006), did not alter conflict performance, suggesting that IL is not required for organizing the avoidance‐risk assessment‐approach sequence measured here. Likewise, lOFC did not affect conflict behavior despite its established involvement in updating action strategies and encoding outcome information (Gremel and Costa 2013; Orsini et al. 2015). One possibility is that lOFC becomes more relevant under conditions requiring flexible updating, contingency shifts, or probabilistic outcomes, whereas SDAmC conflict performance depends more strongly on stable arbitration between an appetitive goal and a learned threat contingency. Similarly, aIC inactivation produced no detectable effects, supporting regional specificity within the insular cortex, and suggesting that anterior insular cortex contributions may be more prominent in tasks emphasizing salience, social valuation, or complex affective appraisal (Rogers‐Carter et al. 2019). Finally, LHb inactivation did not alter conflict behavior despite its prominent role in aversive signaling and avoidance bias (Shabel et al. 2014; Stamatakis and Stuber 2012). LHb may be more critical under conditions involving punishment prediction errors, uncontrollable stressors, or broader state‐dependent negative bias, whereas the instrumental avoidance demands of SDAmC may rely more directly on amygdala‐dependent aversive memory and prefrontal/insular regulation of action selection.

Importantly, these null effects should not be interpreted as evidence that these regions are irrelevant for motivated decision‐making in general, but rather that their inactivation did not alter the specific behavioral measures quantified in SDAmC under the present experimental conditions. By incorporating negative results alongside positive ones, the current dataset provides a sharper constraint on circuit models of motivated conflict and helps distinguish conflict‐specific hubs from regions whose contributions may emerge under different task demands.

### 4.6 Limitations and Future Directions

Several factors frame the interpretation of these findings. First, experiments were conducted in adult male rats, and sex‐dependent mechanisms may modulate conflict processing; this should be addressed given increasing evidence for sex differences in defensive regulation and motivated behavior (Day et al. 2020; Gruene et al. 2015; Orsini and Setlow 2017; Orsini et al. 2016). Second, muscimol/baclofen inactivation provides regional, but not cell‐type‐ or projection‐specific, causal information. The combined use of a GABAA agonist (muscimol) and a GABAB agonist (baclofen) was intended to achieve broad suppression of local neural activity, but this approach does not distinguish among excitatory neurons, inhibitory interneurons, or passing fibers. Thus, the findings identify regions whose activity is necessary for specific SDAmC behavioral effects, but they do not resolve the neuronal populations, afferent inputs, or efferent pathways that mediate these effects; future work using pathway‐selective manipulations will be necessary to determine whether the PL–NAc, pIC–striatal, or amygdala–striatal axes contribute differentially to the behavioral signatures observed here. Third, the SDAmC task captures a specific conflict configuration in which appetitive drive is manipulated by thirst and the threat contingency is learned instrumentally. Future studies varying motivational state, threat controllability, or reinforcement uncertainty may reveal additional region‐specific contributions, including potential roles for structures such as the LHb or OFC under more dynamic decision contexts. The task‐derived movement analyses further argue against generalized hyperlocomotion as the main explanation for the increased approach phenotype. Because distance traveled in SDAmC is constrained by zone occupancy and behavioral strategy, these measures are best interpreted as task‐related movement controls.

## 5 Conclusions

Together, these results identify a constrained set of cortico‐limbic regions that organize reward seeking under threat. PL and pIC constrain conflict‐specific approach through dissociable effects on the coordination of risk assessment and action timing, BLA contributes to the aversive constraint supporting avoidance across motivational states, and NAc supports coherent organization of goal‐directed progression under conflict. By systematically testing both candidate and non‐candidate regions within the same behavioral framework, this work provides causal evidence that motivated conflict behavior is supported by a selective network of hubs rather than a diffuse “emotional” system, offering a clearer foundation for circuit‐level models of approach‐avoidance arbitration.

## Author Contributions

**Rodrigo O. Sierra:** conceptualization, methodology, investigation, formal analysis, writing – original draft, writing – review and editing. **Leticia Ramirez‐Lugo:** conceptualization, methodology, investigation, formal analysis. **Elizabeth Illescas‐Huerta:** conceptualization, methodology, investigation, formal analysis, writing – original draft, writing – review and editing. **Axel R. Bolaños:** investigation, formal analysis. **Francisco Sotres‐Bayon:** conceptualization, methodology, formal analysis, writing – original draft, writing – review and editing.

## Funding

This work was supported by Secretaría de Ciencias Humanidades y Tecnología (SECIHTI; grant CF‐2023‐I‐225) and by Dirección General de Asuntos del Personal Académico, Universidad Nacional Autónoma de México (DGAPA‐UNAM; grants IN214223 and IN226026) to F.S.‐B.

## Conflicts of Interest

The authors declare no conflicts of interest.

## Supporting information


**Figure S1:** Task‐related movement metrics during the SDAmC conflict test. Distance traveled and mean speed were quantified separately for the start, choice, and reward zones in the main regions where inactivation altered conflict behavior: (A) prelimbic cortex (PL), (B) posterior insular cortex (pIC), (C) basolateral amygdala (BLA), and (D) nucleus accumbens (NAc). These task‐derived movement measures were added to evaluate whether the behavioral effects of regional inactivation could be explained by nonspecific locomotor changes. Distance traveled should be interpreted in the context of task performance because movement across zones is constrained by the animal's approach–avoidance strategy. Mean speed provides a complementary estimate of movement output during the session. Inactivation did not produce a generalized increase in mean speed across regions or zones. Data are shown as mean ± SEM with individual data points. Sample sizes were PL (VEH *n* = 13, INACT *n* = 10), pIC (VEH *n* = 9, INACT *n* = 11), BLA (VEH *n* = 5, INACT *n* = 6), and NAc (VEH *n* = 5, INACT *n* = 9). Vehicle and inactivation groups were compared using Mann–Whitney *U* tests. *p < 0.05. PL: reward‐zone distance, *U* = 38.5, *p* = 0.048; reward‐zone speed, *U* = 37.5, *p* = 0.040. pIC: start‐zone distance, *U* = 22.0, *p* = 0.040; choice‐zone distance, *U* = 78.5, *p* = 0.030; reward‐zone distance, *U* = 19.5, *p* = 0.021. BLA: choice‐zone distance, *U* = 27.0, *p* = 0.030; start‐zone speed, *U* = 1.0, *p* = 0.013. NAc: choice‐zone distance, *U* = 40.0, *p* = 0.019; reward‐zone distance, *U* = 3.5, *p* = 0.013. All other comparisons were not significant.

**Data S1:** Completed transparent science questionnaire.

## Data Availability

The data that support the findings of this study are available from the corresponding author upon reasonable request.

## References

[jnr70150-bib-0001] Ambroggi, F. , A. Ishikawa , H. L. Fields , and S. M. Nicola . 2008. “Basolateral Amygdala Neurons Facilitate Reward‐Seeking Behavior by Exciting Nucleus Accumbens Neurons.” Neuron 59, no. 4: 648–661. 10.1016/j.neuron.2008.07.004. 18760700 PMC2603341

[jnr70150-bib-0002] Berridge, K. C. , and T. E. Robinson . 1998. “What Is the Role of Dopamine in Reward: Hedonic Impact, Reward Learning, or Incentive Salience?” Brain Research. Brain Research Reviews 28, no. 3: 309–369. 10.1016/s0165-0173(98)00019-8. 9858756

[jnr70150-bib-0003] Beyeler, A. , P. Namburi , G. F. Glober , et al. 2016. “Divergent Routing of Positive and Negative Information From the Amygdala During Memory Retrieval.” Neuron 90, no. 2: 348–361. 10.1016/j.neuron.2016.03.004. 27041499 PMC4854303

[jnr70150-bib-0004] Blair, H. T. , F. Sotres‐Bayon , M. A. Moita , and J. E. Ledoux . 2005. “The Lateral Amygdala Processes the Value of Conditioned and Unconditioned Aversive Stimuli.” Neuroscience 133, no. 2: 561–569. 10.1016/j.neuroscience.2005.02.043. 15878802

[jnr70150-bib-0005] Bravo‐Rivera, C. , C. Roman‐Ortiz , E. Brignoni‐Perez , F. Sotres‐Bayon , and G. J. Quirk . 2014. “Neural Structures Mediating Expression and Extinction of Platform‐Mediated Avoidance.” Journal of Neuroscience 34, no. 29: 9736–9742. 10.1523/JNEUROSCI.0191-14.2014. 25031411 PMC4099548

[jnr70150-bib-0006] Burgos‐Robles, A. , E. Y. Kimchi , E. M. Izadmehr , et al. 2017. “Amygdala Inputs to Prefrontal Cortex Guide Behavior Amid Conflicting Cues of Reward and Punishment.” Nature Neuroscience 20, no. 6: 824–835. 10.1038/nn.4553. 28436980 PMC5448704

[jnr70150-bib-0007] Burgos‐Robles, A. , I. Vidal‐Gonzalez , and G. J. Quirk . 2009. “Sustained Conditioned Responses in Prelimbic Prefrontal Neurons Are Correlated With Fear Expression and Extinction Failure.” Journal of Neuroscience 29, no. 26: 8474–8482. 10.1523/JNEUROSCI.0378-09.2009. 19571138 PMC2733220

[jnr70150-bib-0008] Calhoon, G. G. , and K. M. Tye . 2015. “Resolving the Neural Circuits of Anxiety.” Nature Neuroscience 18, no. 10: 1394–1404. 10.1038/nn.4101. 26404714 PMC7575249

[jnr70150-bib-0009] Capuzzo, G. , and S. B. Floresco . 2020. “Prelimbic and Infralimbic Prefrontal Regulation of Active and Inhibitory Avoidance and Reward‐Seeking.” Journal of Neuroscience 40, no. 24: 4773–4787. 10.1523/JNEUROSCI.0414-20.2020. 32393535 PMC7294792

[jnr70150-bib-0010] Choi, E. A. , P. Jean‐Richard‐Dit‐Bressel , C. W. G. Clifford , and G. P. McNally . 2019. “Paraventricular Thalamus Controls Behavior During Motivational Conflict.” Journal of Neuroscience 39, no. 25: 4945–4958. 10.1523/JNEUROSCI.2480-18.2019. 30979815 PMC6670259

[jnr70150-bib-0011] Dallerac, G. , M. Graupner , J. Knippenberg , et al. 2017. “Updating Temporal Expectancy of an Aversive Event Engages Striatal Plasticity Under Amygdala Control.” Nature Communications 8: 13920. 10.1038/ncomms13920. PMC5227703 28067224

[jnr70150-bib-0012] Day, H. L. L. , S. Suwansawang , D. M. Halliday , and C. W. Stevenson . 2020. “Sex Differences in Auditory Fear Discrimination Are Associated With Altered Medial Prefrontal Cortex Function.” Scientific Reports 10, no. 1: 6300. 10.1038/s41598-020-63405-w. 32286467 PMC7156682

[jnr70150-bib-0013] Everitt, B. J. , and T. W. Robbins . 2005. “Neural Systems of Reinforcement for Drug Addiction: From Actions to Habits to Compulsion.” Nature Neuroscience 8, no. 11: 1481–1489. 10.1038/nn1579. 16251991

[jnr70150-bib-0014] Floresco, S. B. 2015. “The Nucleus Accumbens: An Interface Between Cognition, Emotion, and Action.” Annual Review of Psychology 66: 25–52. 10.1146/annurev-psych-010213-115159. 25251489

[jnr70150-bib-0015] Friedman, A. , D. Homma , L. G. Gibb , et al. 2015. “A Corticostriatal Path Targeting Striosomes Controls Decision‐Making Under Conflict.” Cell 161, no. 6: 1320–1333. 10.1016/j.cell.2015.04.049. 26027737 PMC4477966

[jnr70150-bib-0016] Gehrlach, D. A. , N. Dolensek , A. S. Klein , et al. 2019. “Aversive State Processing in the Posterior Insular Cortex.” Nature Neuroscience 22, no. 9: 1424–1437. 10.1038/s41593-019-0469-1. 31455886

[jnr70150-bib-0017] Gremel, C. M. , and R. M. Costa . 2013. “Orbitofrontal and Striatal Circuits Dynamically Encode the Shift Between Goal‐Directed and Habitual Actions.” Nature Communications 4: 2264. 10.1038/ncomms3264. PMC4026062 23921250

[jnr70150-bib-0018] Gruene, T. M. , E. Roberts , V. Thomas , A. Ronzio , and R. M. Shansky . 2015. “Sex‐Specific Neuroanatomical Correlates of Fear Expression in Prefrontal‐Amygdala Circuits.” Biological Psychiatry 78, no. 3: 186–193. 10.1016/j.biopsych.2014.11.014. 25579850 PMC4449316

[jnr70150-bib-0019] Hernandez‐Jaramillo, A. , E. Illescas‐Huerta , and F. Sotres‐Bayon . 2024. “Ventral Pallidum and Amygdala Cooperate to Restrain Reward Approach Under Threat.” Journal of Neuroscience 44, no. 23. 10.1523/JNEUROSCI.2327-23.2024. PMC11154850 38631914

[jnr70150-bib-0020] Illescas‐Huerta, E. , L. Ramirez‐Lugo , R. O. Sierra , J. A. Quillfeldt , and F. Sotres‐Bayon . 2021. “Conflict Test Battery for Studying the Act of Facing Threats in Pursuit of Rewards.” Frontiers in Neuroscience 15: 645769. 10.3389/fnins.2021.645769. 34017234 PMC8129192

[jnr70150-bib-0021] Janak, P. H. , and K. M. Tye . 2015. “From Circuits to Behaviour in the Amygdala.” Nature 517, no. 7534: 284–292. 10.1038/nature14188. 25592533 PMC4565157

[jnr70150-bib-0022] LeDoux, J. E. 2000. “Emotion Circuits in the Brain.” Annual Review of Neuroscience 23: 155–184. 10.1146/annurev.neuro.23.1.155. 10845062

[jnr70150-bib-0023] Livneh, Y. , R. N. Ramesh , C. R. Burgess , et al. 2017. “Homeostatic Circuits Selectively Gate Food Cue Responses in Insular Cortex.” Nature 546, no. 7660: 611–616. 10.1038/nature22375. 28614299 PMC5577930

[jnr70150-bib-0024] Maren, S. 2001. “Neurobiology of Pavlovian Fear Conditioning.” Annual Review of Neuroscience 24: 897–931. 10.1146/annurev.neuro.24.1.897. 11520922

[jnr70150-bib-0025] McNaughton, N. , and P. J. Corr . 2014. “Approach, Avoidance, and Their Conflict: The Problem of Anchoring.” Frontiers in Systems Neuroscience 8: 124. 10.3389/fnsys.2014.00124. 25071476 PMC4079981

[jnr70150-bib-0026] Miller, N. E. 1944. “Experimental Studies of Conflict.” In Personality and the Behavior Disorders, edited by J. M. Hunt , 431–465. Ronald Press.

[jnr70150-bib-0027] Moorman, D. E. , and G. Aston‐Jones . 2015. “Prefrontal Neurons Encode Context‐Based Response Execution and Inhibition in Reward Seeking and Extinction.” Proceedings of the National Academy of Sciences of the United States of America 112, no. 30: 9472–9477. 10.1073/pnas.1507611112. 26170333 PMC4522823

[jnr70150-bib-0028] Moscarello, J. M. , and J. E. LeDoux . 2013. “Active Avoidance Learning Requires Prefrontal Suppression of Amygdala‐Mediated Defensive Reactions.” Journal of Neuroscience 33, no. 9: 3815–3823. 10.1523/JNEUROSCI.2596-12.2013. 23447593 PMC3607300

[jnr70150-bib-0029] Namburi, P. , A. Beyeler , S. Yorozu , et al. 2015. “A Circuit Mechanism for Differentiating Positive and Negative Associations.” Nature 520, no. 7549: 675–678. 10.1038/nature14366. 25925480 PMC4418228

[jnr70150-bib-0030] Orsini, C. A. , D. E. Moorman , J. W. Young , B. Setlow , and S. B. Floresco . 2015. “Neural Mechanisms Regulating Different Forms of Risk‐Related Decision‐Making: Insights From Animal Models.” Neuroscience and Biobehavioral Reviews 58: 147–167. 10.1016/j.neubiorev.2015.04.009. 26072028 PMC7913606

[jnr70150-bib-0031] Orsini, C. A. , and B. Setlow . 2017. “Sex Differences in Animal Models of Decision Making.” Journal of Neuroscience Research 95, no. 1–2: 260–269. 10.1002/jnr.23810. 27870448 PMC5120608

[jnr70150-bib-0032] Orsini, C. A. , M. L. Willis , R. J. Gilbert , J. L. Bizon , and B. Setlow . 2016. “Sex Differences in a Rat Model of Risky Decision Making.” Behavioral Neuroscience 130, no. 1: 50–61. 10.1037/bne0000111. 26653713 PMC4738105

[jnr70150-bib-0033] Pape, H. C. , and D. Pare . 2010. “Plastic Synaptic Networks of the Amygdala for the Acquisition, Expression, and Extinction of Conditioned Fear.” Physiological Reviews 90, no. 2: 419–463. 10.1152/physrev.00037.2009. 20393190 PMC2856122

[jnr70150-bib-0034] Parkes, S. L. , L. A. Bradfield , and B. W. Balleine . 2015. “Interaction of Insular Cortex and Ventral Striatum Mediates the Effect of Incentive Memory on Choice Between Goal‐Directed Actions.” Journal of Neuroscience 35, no. 16: 6464–6471. 10.1523/JNEUROSCI.4153-14.2015. 25904797 PMC4405558

[jnr70150-bib-0035] Paterson, N. E. , and T. Hanania . 2010. “The Modified Geller‐Seifter Test in Rats Was Insensitive to GABAB Receptor Positive Modulation or Blockade, or 5‐HT1A Receptor Activation.” Behavioural Brain Research 208, no. 1: 258–264. 10.1016/j.bbr.2009.12.006. 20006648

[jnr70150-bib-0036] Paxinos, G. , and C. Watson . 1998. The Rat Brain in Stereotaxic Coordinates. Academic Press. 10.1016/0165-0270(80)90021-7 6110810

[jnr70150-bib-0039] Pickens, C. L. , M. P. Saddoris , B. Setlow , M. Gallagher , P. C. Holland , and G. Schoenbaum . 2003. “Different Roles for Orbitofrontal Cortex and Basolateral Amygdala in a Reinforcer Devaluation Task.” Journal of Neuroscience 23, no. 35: 11078–11084. 10.1523/JNEUROSCI.23-35-11078.2003. 14657165 PMC6741041

[jnr70150-bib-0040] Ramirez‐Lugo, L. , A. Penas‐Rincon , S. Angeles‐Duran , and F. Sotres‐Bayon . 2016. “Choice Behavior Guided by Learned, but Not Innate, Taste Aversion Recruits the Orbitofrontal Cortex.” Journal of Neuroscience 36, no. 41: 10574–10583. 10.1523/JNEUROSCI.0796-16.2016. 27733609 PMC6601934

[jnr70150-bib-0041] Rogers‐Carter, M. M. , A. Djerdjaj , K. B. Gribbons , J. A. Varela , and J. P. Christianson . 2019. “Insular Cortex Projections to Nucleus Accumbens Core Mediate Social Approach to Stressed Juvenile Rats.” Journal of Neuroscience 39, no. 44: 8717–8729. 10.1523/JNEUROSCI.0316-19.2019. 31591155 PMC6820210

[jnr70150-bib-0042] Salamone, J. D. , and M. Correa . 2012. “The Mysterious Motivational Functions of Mesolimbic Dopamine.” Neuron 76, no. 3: 470–485. 10.1016/j.neuron.2012.10.021. 23141060 PMC4450094

[jnr70150-bib-0043] Salamone, J. D. , M. Pardo , S. E. Yohn , L. Lopez‐Cruz , N. SanMiguel , and M. Correa . 2016. “Mesolimbic Dopamine and the Regulation of Motivated Behavior.” Current Topics in Behavioral Neurosciences 27: 231–257. 10.1007/7854_2015_383. 26323245

[jnr70150-bib-0044] Shabel, S. J. , C. D. Proulx , J. Piriz , and R. Malinow . 2014. “Mood Regulation. GABA/Glutamate Co‐Release Controls Habenula Output and Is Modified by Antidepressant Treatment.” Science 345, no. 6203: 1494–1498. 10.1126/science.1250469. 25237099 PMC4305433

[jnr70150-bib-0045] Shi, T. , S. Feng , M. Wei , and W. Zhou . 2020. “Role of the Anterior Agranular Insular Cortex in the Modulation of Fear and Anxiety.” Brain Research Bulletin 155: 174–183. 10.1016/j.brainresbull.2019.12.003. 31816406

[jnr70150-bib-0046] Shi, W. , Y. Fu , T. Shi , and W. Zhou . 2022. “Different Synaptic Plasticity After Physiological and Psychological Stress in the Anterior Insular Cortex in an Observational Fear Mouse Model.” Frontiers in Synaptic Neuroscience 14: 851015. 10.3389/fnsyn.2022.851015. 35645764 PMC9132225

[jnr70150-bib-0047] Sierra‐Mercado, D. , N. Padilla‐Coreano , and G. J. Quirk . 2011. “Dissociable Roles of Prelimbic and Infralimbic Cortices, Ventral Hippocampus, and Basolateral Amygdala in the Expression and Extinction of Conditioned Fear.” Neuropsychopharmacology 36, no. 2: 529–538. 10.1038/npp.2010.184. 20962768 PMC3005957

[jnr70150-bib-0048] Sotres‐Bayon, F. , and G. J. Quirk . 2010. “Prefrontal Control of Fear: More Than Just Extinction.” Current Opinion in Neurobiology 20, no. 2: 231–235. 10.1016/j.conb.2010.02.005. 20303254 PMC2878722

[jnr70150-bib-0049] Sotres‐Bayon, F. , D. Sierra‐Mercado , E. Pardilla‐Delgado , and G. J. Quirk . 2012. “Gating of Fear in Prelimbic Cortex by Hippocampal and Amygdala Inputs.” Neuron 76, no. 4: 804–812. 10.1016/j.neuron.2012.09.028. 23177964 PMC3508462

[jnr70150-bib-0050] Stamatakis, A. M. , and G. D. Stuber . 2012. “Activation of Lateral Habenula Inputs to the Ventral Midbrain Promotes Behavioral Avoidance.” Nature Neuroscience 15, no. 8: 1105–1107. 10.1038/nn.3145. 22729176 PMC3411914

[jnr70150-bib-0051] Stopper, C. M. , and S. B. Floresco . 2014. “What's Better for Me? Fundamental Role for Lateral Habenula in Promoting Subjective Decision Biases.” Nature Neuroscience 17, no. 1: 33–35. 10.1038/nn.3587. 24270185 PMC4974073

[jnr70150-bib-0052] Stuber, G. D. , D. R. Sparta , A. M. Stamatakis , et al. 2011. “Excitatory Transmission From the Amygdala to Nucleus Accumbens Facilitates Reward Seeking.” Nature 475, no. 7356: 377–380. 10.1038/nature10194. 21716290 PMC3775282

[jnr70150-bib-0053] Tovote, P. , J. P. Fadok , and A. Luthi . 2015. “Neuronal Circuits for Fear and Anxiety.” Nature Reviews. Neuroscience 16, no. 6: 317–331. 10.1038/nrn3945. 25991441

[jnr70150-bib-0054] Tye, K. M. 2018. “Neural Circuit Motifs in Valence Processing.” Neuron 100, no. 2: 436–452. 10.1016/j.neuron.2018.10.001. 30359607 PMC6590698

[jnr70150-bib-0055] Tye, K. M. , R. Prakash , S. Y. Kim , et al. 2011. “Amygdala Circuitry Mediating Reversible and Bidirectional Control of Anxiety.” Nature 471, no. 7338: 358–362. 10.1038/nature09820. 21389985 PMC3154022

[jnr70150-bib-0056] Velazquez‐Hernandez, G. , and F. Sotres‐Bayon . 2021. “Lateral Habenula Mediates Defensive Responses Only When Threat and Safety Memories Are in Conflict.” eNeuro 8, no. 2. 10.1523/ENEURO.0482-20.2021. PMC8059882 33712440

[jnr70150-bib-0057] Vidal‐Gonzalez, I. , B. Vidal‐Gonzalez , S. L. Rauch , and G. J. Quirk . 2006. “Microstimulation Reveals Opposing Influences of Prelimbic and Infralimbic Cortex on the Expression of Conditioned Fear.” Learning & Memory 13, no. 6: 728–733. 10.1101/lm.306106. 17142302 PMC1783626

[jnr70150-bib-0058] Vogel, J. R. , B. Beer , and D. E. Clody . 1971. “A Simple and Reliable Conflict Procedure for Testing Anti‐Anxiety Agents.” Psychopharmacologia 21, no. 1: 1–7. 10.1007/BF00403989. 5105868

[jnr70150-bib-0059] Wassum, K. M. , and A. Izquierdo . 2015. “The Basolateral Amygdala in Reward Learning and Addiction.” Neuroscience and Biobehavioral Reviews 57: 271–283. 10.1016/j.neubiorev.2015.08.017. 26341938 PMC4681295

